# NOTCH Signaling in T-Cell-Mediated Anti-Tumor Immunity and T-Cell-Based Immunotherapies

**DOI:** 10.3389/fimmu.2018.01718

**Published:** 2018-08-20

**Authors:** Michelle A. Kelliher, Justine E. Roderick

**Affiliations:** Department of Molecular, Cell and Cancer Biology, University of Massachusetts Medical School, Worcester, MA, United States

**Keywords:** NOTCH, T lymphocytes, cancer, immunotherapy, anti-tumor response

## Abstract

The NOTCH (1–4) family of receptors are highly conserved and are critical in regulating many developmental processes and in the maintenance of tissue homeostasis. Our laboratory and numerous others have demonstrated that aberrant NOTCH signaling is oncogenic in several different cancer types. Conversely, there is also evidence that NOTCH can also function as a tumor suppressor. In addition to playing an essential role in tumor development, NOTCH receptors regulate T-cell development, maintenance, and activation. Recent studies have determined that NOTCH signaling is required for optimal T-cell-mediated anti-tumor immunity. Consequently, tumor cells and the tumor microenvironment have acquired mechanisms to suppress NOTCH signaling to evade T-cell-mediated killing. Tumor-mediated suppression of NOTCH signaling in T-cells can be overcome by systemic administration of NOTCH agonistic antibodies and ligands or proteasome inhibitors, resulting in sustained NOTCH signaling and T-cell activation. In addition, NOTCH receptors and ligands are being utilized to improve the generation and specificity of T-cells for adoptive transplant immunotherapies. In this review, we will summarize the role(s) of NOTCH signaling in T-cell anti-tumor immunity as well as TCR- and chimeric antigen receptor-based immunotherapies.

## Introduction

There are four NOTCH receptors (NOTCH1–4) in mammals, which are ubiquitously expressed. Activation of the NOTCH signaling occurs after engagement of a NOTCH receptor with one of its membrane bound Delta-like ligands 1,3,4 (DLL1, DLL3, DLL4) or Jagged ligands 1,2. In some contexts, NOTCH can become activated through ligand-independent mechanism(s) leading to a variety of human diseases (1). After ligand engagement NOTCH undergoes a series of proteolytic cleavages, resulting in an activated NOTCH intracellular domain (NICD), which translocates into the nucleus to activate gene transcription. Given that NOTCH signaling is critical in regulating cell fate decisions in many tissue types, it is not surprising that NOTCH activity is deregulated in several malignancies (2–4). The first evidence for the involvement of NOTCH signaling in cancer was discovered in T-cell acute lymphoblastic leukemia (T-ALL), where activating mutations were identified in NOTCH1 (5). Our laboratory showed that oncogenic NOTCH1 regulates MYC expression and leukemia-initiating cell activity and demonstrated the efficacy of NOTCH1 inhibitors in pre-clinical T-ALL models (6–9). Activating mutations in *NOTCH1* have also been identified in chronic lymphocytic leukemia, non-small cell lung carcinoma, and translocations involving NOTCH1/2 in patients with triple negative breast cancer (10–13). While mutations in NOTCH receptors are rare in other tumor types, NOTCH is aberrantly activated in several malignancies, including colorectal and pancreatic cancer, melanoma, adenocystic carcinoma, and medulloblastoma through a variety of mechanisms (2, 4). Conversely, loss of function mutations in *NOTCH1/2/3* have also been identified suggesting NOTCH can also function as a tumor suppressor (2, 3).

While progress has been made in how NOTCH signaling contributes to malignant transformation, the role of NOTCH activity in anti-tumor immune responses is less clear. While several cell types contribute to anti-tumor responses, CD4 T-helper 1 (TH1) cells and CD8 cytotoxic T-lymphocytes (CTL), are critical in mediating anti-tumor immunity due to their ability to recognize tumor antigens and mediate tumor killing. Several studies have shown that NOTCH is required for activation and effector function of CD4 and CD8 T-cells (14). Tumor cells can dampen T-cell responses by producing immunosuppressive cytokines, expressing inhibitory ligands, and recruiting immunosuppressive myeloid and lymphoid cells into the tumor microenvironment (15). Given that NOTCH is required for T-cell activation and effector function it is reasonable to hypothesize that NOTCH contributes to T-cell anti-tumor responses and that tumor cells may evade T-cell mediated killing by suppressing NOTCH activation. Consistent with this hypothesis, new data suggest that NOTCH activation is suppressed in tumor-infiltrating T-cells and that NOTCH re-activation induces potent anti-tumor T-cell responses in mouse cancer models (16–20).

Adoptive transplants of tumor antigen-specific T-cells is one immunotherapy used to overcome the limitations of endogenous T-cells and enhance anti-tumor responses. Tumor antigen-specific T-cells are either isolated from the tumor site or engineered with synthetic T-cell receptors (sTCRs) or chimeric antigen receptors (CARs) specific for tumor antigens (21, 22). Recently, NOTCH signaling has been utilized to improve the generation and efficacy of adoptive T-cell therapies (ACT) (23, 24). Furthermore, newly developed synthetic NOTCH receptors (synNOTCH) have been engineered to enhance the specificity of CAR T-cells (25–27). These studies highlight the importance of studying NOTCH responses in T-cell-mediated anti-tumor immunity in order to design more effective T-cell-based immunotherapies.

## NOTCH Signaling is Required for T-Cell Activation and Effector Function

NOTCH signaling has been extensively studied in T-cell development, activation, and effector function. Upon TCR-stimulation naïve CD4 T-cells differentiate into multiple subsets of T-helper (TH) cells (14, 28). TH subsets are designed to recognize and fight distinct types of infection and are characterized by their specific cytokine profile. NOTCH activation has been shown to play a role in the differentiation of TH1, TH2, TH9, TH17, T-regulatory cells, and follicular TH cells (14, 28). TH1 cells mediate anti-tumor responses in conjunction with CTLs. Genetic deletion or pharmacologic inhibition of NOTCH1 signaling with gamma-secretase inhibitors (GSIs) decreases the numbers of activated TH1 cells *in vitro* and in mouse models of TH1-driven autoimmune disease (29, 30). NOTCH directly stimulates the transcription of the TH1 master transcriptional regulator T-BET (*TBX21*) as well as the TH1 signature cytokine interferon-gamma (*IFNγ*) (29–31).

CD8 naïve T-cells differentiate into CTLs upon early TCR stimulation, and then terminal effector cells or memory precursor cells (14). Recent evidence shows that conditional deletion of *Notch1* or inhibition of NOTCH signaling with GSIs diminishes the production of CTL effector molecules, including IFNγ, tumor necrosis factor alpha, granzyme B, and perforin, as well as a reduction in the CD8 transcription factors T-BET and eomesodermin (EOMES) (32–36). In addition to playing a role in activating effector T-cells NOTCH is also important in the maintenance and generation of memory T-cells (35, 37). While these studies provide compelling evidence that NOTCH signaling regulates T-cell effector activation, it remains unclear how NOTCH dictates such a multitude of responses in T-cells. Data from several studies suggest that NOTCH ligands may dictate T-cell effector responses.

## NOTCH Ligands Dictate T-Cell Fate

NOTCH ligands have been shown to have diverse effects on T-cell effector function. In CD4 T-cells, activation of the TCR in the presence of DLL1/4 skews toward a TH1 fate and inhibits TH2 differentiation (38, 39). Conversely, Jagged1/2 ligands may be important for TH2 differentiation, but appear to have no role in TH1 differentiation (38, 39). The role of DLL1 in CD8 T-cell activation and differentiation is unclear (38, 39). One study found that DLL1 overexpression in dendritic cells results in increased levels of granzyme-B expression in alloantigen stimulated CD8 T-cells (32). However, a prior study reported that CD8 T-cells stimulated with DLL1 and alloantigens resulted in decreased IFN-γ production and increased IL-10 production, suggesting a suppressive role for DLL1 in CD8 activation (40). Additional studies are needed to clarify the effects of DLL1 and other NOTCH ligands on the activation and effector function of CTLs.

These studies suggest that T-cell effector function mediated by NOTCH is determined by the stimulating ligand, this is further supported by data demonstrating that ligand expression on antigen-presenting cells (APC) is dictated by the engaging stimulus. For example, APC exposed to allergens upregulate Jagged1/2 expression inducing a TH2 response whereas viral infection stimulates DLL1/4 expression on APC and a TH1 response (41, 42). However, some studies demonstrate normal T-cell polarization and effector function in the absence of NOTCH ligands, favoring a model in which NOTCH enhances T-cell activation and proliferation, however, cytokines instruct T-cell fate (39). Understanding how NOTCH ligands dictate effector function will be critical to maximize the therapeutic potential of NOTCH-based immunotherapies.

## Tumor Cells and Their Microenvironment Suppress the Expression of NOTCH Receptors and Ligands

Full-length NOTCH receptors are normally expressed on naïve mouse T-cells and activated in response to antigen; however, T-cells isolated from tumor bearing mice have decreased expression of NOTCH (1–4), (18, 19). Consistent with this reduction in NOTCH levels, significant decreases in NOTCH target genes (*Deltex1, Hey1*, and *Hes1*) are also observed in tumor-associated T-cells (19), suggesting that tumor-associated T-cells have repressed NOTCH signaling and potentially decreased effector function.

Reduction in NOTCH1/2 levels was found to be mediated in part by tumor-infiltrating myeloid-derived suppressor cells (MDSCs) (18). MDSCs are a heterogeneous population of immature myeloid cells that are recruited to sites of inflammation and the tumor microenvironment to prevent immune-mediated damage (43). MDSCs are recruited by multiple factors including vascular endothelial growth factor (VEGF), IL-1β, and IL-6 (44). Coculturing of MDSC with activated T-cells reduced the expression of full length and intracellular NOTCH1/2 (18). MDSC isolated from cancer patients have been shown to suppress T-cell activation (45, 46), however, whether MDSC suppress *via* effects on NOTCH signaling is not known.

In addition to reducing NOTCH1/2 levels, reductions in NOTCH ligand expression on T-cells and other immune cells has also been observed in murine tumor models (16, 19). Reduced expression of DLL1/4 in the bone marrow of tumor bearing mice inversely correlated with increased VEGF levels in one study (16). VEGF has been shown to potentiate T-cell anti-tumor responses, suggesting that expression of this growth factor by cancer cells may inhibit T-cell responses by downregulating DLL1/4 (47). MDSC isolated from the tumor site have decreased DLL1/4 and increased Jagged1/2 expression (18). Given that DLL1/4 induce TH1 and CTL effector function, this could be an additional mechanism, whereby the tumor microenvironment impairs/disables NOTCH signaling. While these studies demonstrate that NOTCH activity is impaired in tumor-infiltrating T-cells in mouse cancer models, precisely how NOTCH receptor/ligands are downregulated is unclear. Furthermore, there is as yet, no direct evidence that NOTCH signaling is impaired in T-cells from cancer patients.

## Activation of NOTCH Receptors and Their Ligands Increases T-Cell-Mediated Anti-Tumor Response

Conditional activation of NOTCH1/2 in CD8 T-cells induces a robust and sustained anti-tumor response, resulting in increased IFNγ production and reduced tumor burden (18, 20). Similarly, treatment of tumor bearing mice with an agonistic NOTCH2 antibody enhanced CD8 T-cell cytotoxicity and reduced tumor size (20). Consistent with this finding, conditional deletion of *Notch2* in CD8 T-cells potentiated tumor growth in mice and reduced overall survival (20).

Constitutive expression of DLL1 on bone marrow and dendritic cells was also reported to enhance T-cell infiltration into tumors, suppress tumor growth and increase the survival of mice transplanted with murine tumor cell lines [Lewis Lung Carcinoma (LLC), D459 Fibrosarcoma, and EL4 T cell Lymphoma] (16, 20). Increased DLL1 but not Jagged2 expression on dendritic cells stimulated T-cell cytotoxicity and increased IFN-γ levels (20). Moreover, therapeutic administration of a multivalent, clustered form of DLL1 (c-DLL1) arrested tumor growth and prolonged survival of mice transplanted with LLCs or D459 tumor cells (16, 17). The c-DLL1 was shown to bind and activate NOTCH (1–4), resulting in increased NOTCH target gene expression (16, 17). Administration of c-DLL1 stimulated IFN-γ production and increased tumor-infiltrating antigen-specific T-cells (16, 17). Tumor regression in c-DLL1 treated mice appears to be T-cell mediated, since c-DLL1 treatment had no effect on tumor growth in *Rag1*^−/−^ recipients or in mice treated with anti-CD8 antibody (16). Furthermore, adoptive transfer of tumor antigen-specific T-cells from c-DLL1-treated mice were sufficient to attenuate tumor growth in immunocompromised NOD-SCID mice (17).

The proteasome inhibitor bortezomib was shown to enhance T-cell-mediated anti-tumor responses in part by restoration of NOTCH receptors and ligand mRNA expression (19). Bortezomib treatment led to increased expression of CD25, CD44, IFNγ, and granzyme B in CD8^+^ T-cells isolated from mice engrafted with cancer cell lines (19, 48). Combination treatments consisting of bortezomib and adoptive T-cell transfer reduced tumor burden and prolonged survival in human renal carcinoma xenografts (48). Whether bortezomib treatment regulates NOTCH activity directly or if these effects are secondary is unknown. Together these studies support the concept that activating NOTCH enhances T-cell anti-tumor immunity and prolongs tumor-free survival. While the development of NOTCH agonist antibodies and c-DLL1 therapies appear to be a promising approach to enhance T-cell anti-tumor immunity, the potential effects on NOTCH driven malignancies needs to be considered.

## Current Challenges in ACT

Adoptive T-cell therapies involves the generation of tumor antigen-specific CTLs *in vitro*, which are then infused back into the patient where they kill tumor cells. Tumor-specific T-cells are generated by selection and expansion of tumor-infiltrating lymphocytes (TIL), or by transduction of sTCR or CAR (21, 22). ACT using TILs has been a successful treatment option for melanoma, however, this approach could only be used on patients whose T-cells could be isolated and cultured (49, 50). CAR T-cell therapies have yielded exceptional clinical results in B-ALL (51–53), but identification of tumor-specific antigens is needed in order to expand CAR T-cell therapies to additional malignancies. Both approaches need improvement because the generation of TIL and CAR T-cells is time consuming and T-cell numbers are limiting. Furthermore, while the T-cells used for ACT have enhanced tumor antigen recognition, they are still susceptible to immunosuppressive factors in the tumor microenvironment.

## NOTCH Ligands in T-Cell-Based Immunotherapies

Generation of CAR-specific T-cells from induced pluripotent stem cells (iPSC) from cancer patients is one approach currently being utilized to overcome limited numbers of patient T-cells (24). Using this approach iPSC are differentiated into T-cells by culturing on stroma expressing the NOTCH ligand DLL1 (24). Similar approaches have been used to generate CAR T-cells from hematopoietic stem cells (54). Researchers have also used pluripotency and reprogramming factors to expand human tumor-specific T-cells (55, 56). While this strategy produces unlimited tumor-specific CTLs, the TCR repertoires are often limited. To overcome this obstacle, investigators have begun to test the efficacy of T-stem cell memory (T_SCM_) cells in adoptive T-cell transplants. T_SCM_ cells have the ability to function as memory T-cells by responding rapidly to antigen, however, they are not terminally differentiated and therefore possess an enhanced capacity for self-renewal and proliferation (57). T_SCM_ cells have been characterized in mice and humans and found to persist years after primary infection or vaccination. The current model to generate T_SCM_ cells is by stimulating naïve T-cells in the presence of Wnt3A or inhibitors of glycogen synthase kinase-3b (57). Adoptive T-cell transplants with CAR T-cells generated from T_SCM_ cells results in more potent anti-tumor responses than CAR T-cells generated from other T-cell types (57). Recent work by Kondo et al. exploit NOTCH pathway activation to generate T_SCM_ cells from activated mouse and human T-cells referred to as iT_SCM_ cells (23). iT_SCM_ cells re-capitulate the features of T_SCM_ cells including rapid response to antigen re-stimulation and increased self-renewal capacity. iT_SCM_ cells also exhibit decreased expression of the T-cell inhibitory receptors programmed cell death-1 (PD-1) and cytotoxic-T-lymphocyte-associated protein 4 (CTLA-4), allowing for enhanced survival and activation in the tumor microenvironment (23). Unlike traditional T_SCM_ cells generated from naïve T-cells, iT_SCM_ cells are derived from activated T-cells and therefore could be generated from TILs, eliminating the need for transduction with sTCRs or CARs. These alternative methods to generate T-cells for ACT may provide greater anti-tumor immunity by increasing T-cell longevity and yield.

## Synthetic NOTCH Receptors Generate Potent CAR Specific T-Cells

While current methods have markedly enhanced anti-tumor reactivity, CAR T-cells are still restricted to endogenous T-cell responses have limited capabilities to overcome the immunosuppressive microenvironment. To overcome this, researchers generated CAR T-cells with synthetic NOTCH receptors (synNOTCH), which allow for specific cytotoxic responses (26, 27). NOTCH receptors are single pass transmembrane proteins composed of an extracellular ligand-binding domain, a transmembrane region, and an intracellular signaling domain. synNOTCH receptors contain the transmembrane domain, however, they have synthetic extracellular ligand domains and intracellular transcriptional domains (26, 27). In recent work by Roybal et al., human T-cells were engineered to express synNOTCH receptors, where the extracellular ligand domain of NOTCH was replaced with CARs targeting tumor antigens, CD19 or HER2 (27). Following CAR engagement, the synNOTCH receptor undergoes transmembrane cleavage, releasing the synthetic NICD. NICD then translocates to the nucleus to activate gene transcription. Unlike normal NICD which recognizes and binds CBF1/RBP-Jkappa sites, synthetic NICD is replaced with an intracellular transcription activation domain (Gal4-VP64 or tTA) that in turn drives a distinct reporter expressed in the synNOTCH expressing cell (26, 27). synNOTCH receptors have been engineered to drive the expression of several cytotoxic factors that enhance T-cell anti-tumor responses, including expression of the death ligand TRAIL, the cytokine IL-12, and the transcription factor T-BET. In addition, synNOTCH receptors can drive the production of antibodies to PD-1 and CTLA-4 to overcome inhibitory ligand expression by cancer cells or express IL-10 and PD-L1 to reduce inflammation generated by enhanced T-cell cytotoxicity. synNOTCH-engineered T-cells have shown efficacy in conventional humanized xenograft models (27). Using these synNOTCH receptors to customize CAR T-cell responses will enhance anti-tumor activity, and armor the T-cells against the immune suppression mediated by the tumor microenvironment.

## Conclusion/Future Perspectives

Activation of T-cell effector function in an immunosuppressive microenvironment is a critical component of effective T-cell-mediated anti-tumor immunity. Tumor cells and their microenvironment suppress T-cell responses in part by repressing NOTCH receptors and ligands and consequently T-cell effector function. While in-depth characterization of tumor cells has led to the development of targeted therapies, characterization of tumor-infiltrating T-cells from patients is still lacking. Several studies have begun to establish gene signatures that represent a variety of immune populations and demonstrated that these signatures can be predictive of clinical outcome and response to immune therapy (58, 59). A similar approach could be taken to determine if NOTCH receptors and ligands are suppressed in T-cells isolated from cancer patients. Therapies that activate/maintain NOTCH signaling were shown to improve T-cell-mediated tumor clearance, prolonging the survival of tumor bearing mice. However, the efficacy and safety of this approach in patients remains unclear.

NOTCH ligands may also serve as tools to improve the generation and efficacy of T-cells used for ACT. T_SCM_ cells may overcome the obstacles currently facing ACT, including increasing anti-tumor responses and decreasing immunosuppression. The use of synNOTCH CAR T-cells is particularly intriguing as the cytotoxic response of these cells can be tailored to provide an enhanced and specific anti-tumor response. Future studies examining combinations of synNOTCH T-cells on the anti-tumor immune responses and their effects on endogenous tumor-infiltrating T-cells should provide insight.

While these findings highlight the exciting potential to improve T-cell-based immunotherapies, there are still many questions regarding the clinical relevance and application of these approaches. In addition, the safety and efficacy of these NOTCH strategies need to be evaluated to ensure that sustained NOTCH activation does not result in leukemic transformation or potentiate tumor growth. One major limitation in accomplishing these goals is the lack of a primary derived xenograft mouse model with a humanized immune system. Continued research will provide a better understanding as to how NOTCH signaling contributes to T-cell anti-tumor responses and uncover new approaches to improve T-cell-based immunotherapies.

## Author Contributions

JR wrote the manuscript with help from MK.

## Conflict of Interest Statement

The authors declare that the research was conducted in the absence of any commercial or financial relationships that could be construed as a potential conflict of interest. The handling Editor declared a shared affiliation, though no other collaboration, with the authors.
